# NeMeSys: a biological resource for narrowing the gap between sequence and function in the human pathogen *Neisseria meningitidis*

**DOI:** 10.1186/gb-2009-10-10-r110

**Published:** 2009-10-09

**Authors:** Christophe Rusniok, David Vallenet, Stéphanie Floquet, Helen Ewles, Coralie Mouzé-Soulama, Daniel Brown, Aurélie Lajus, Carmen Buchrieser, Claudine Médigue, Philippe Glaser, Vladimir Pelicic

**Affiliations:** 1 Génomique des Microorganismes Pathogènes, Institut Pasteur, rue du Dr Roux, Paris, 75015, France; 2 Génomique Métabolique, CNRS UMR8030, Laboratoire de Génomique Comparative, CEA-Institut de Génomique-Génoscope, rue Gaston Crémieux, Evry, 91057, France; 3 U570 INSERM, Faculté de Médecine René Descartes-Paris 5, rue de Vaugirard, Paris, 75015, France; 4 Department of Microbiology, CMMI, Imperial College London, Armstrong Road, London, SW7 2AZ, UK; 5 Current address: Biologie des Bactéries Intracellulaires, Institut Pasteur, rue du Dr Roux, Paris, 75015, France; 6 Current address: Mutabilis, Parc Biocitech, avenue Gaston Roussel, Romainville, 93230, France; 7 Current address: FAB pharma, rue Saint Honoré, Paris, 75001, France

## Abstract

**Background:**

Genome sequences, now available for most pathogens, hold promise for the rational design of new therapies. However, biological resources for genome-scale identification of gene function (notably genes involved in pathogenesis) and/or genes essential for cell viability, which are necessary to achieve this goal, are often sorely lacking. This holds true for *Neisseria meningitidis*, one of the most feared human bacterial pathogens that causes meningitis and septicemia.

**Results:**

By determining and manually annotating the complete genome sequence of a serogroup C clinical isolate of *N. meningitidis *(strain 8013) and assembling a library of defined mutants in up to 60% of its non-essential genes, we have created NeMeSys, a biological resource for *Neisseria meningitidis *systematic functional analysis. To further enhance the versatility of this toolbox, we have manually (re)annotated eight publicly available *Neisseria *genome sequences and stored all these data in a publicly accessible online database. The potential of NeMeSys for narrowing the gap between sequence and function is illustrated in several ways, notably by performing a functional genomics analysis of the biogenesis of type IV pili, one of the most widespread virulence factors in bacteria, and by identifying through comparative genomics a complete biochemical pathway (for sulfur metabolism) that may potentially be important for nasopharyngeal colonization.

**Conclusions:**

By improving our capacity to understand gene function in an important human pathogen, NeMeSys is expected to contribute to the ongoing efforts aimed at understanding a prokaryotic cell comprehensively and eventually to the design of new therapies.

## Abstract 2

The genome of a clinical isolate of Neisseria meningitidis is described.  This and other reannotated Neisseria genomes are compiled in a database.

## Background

By revealing complete repertoires of genes, genome sequences provide the key to a better and eventually global understanding of the biology of living organisms. It is widely accepted that this will have important consequences on human health and economics by leading to the rational design of novel therapies against pathogens infecting humans, livestock or crops [1]. For example, identifying genes essential for cell viability or pathogenesis would uncover targets for new antibiotics or drugs that selectively interfer with virulence mechanisms of pathogenic species, respectively. The major obstacle to this is the fact that hundreds of predicted coding sequences (CDSs) in every genome remain uncharacterized. Unraveling gene function on such a large scale requires suitable biological resources, which are lacking in most species.

As shown in *Saccharomyces cerevisiae*, the model organism for genomics, the most valuable toolbox for determining gene function on a genome scale is likely to be a comprehensive archived collection of mutants [2]. In bacteria, archived collections of mutants containing mutations in most or all non-essential genes have been constructed by systematic targeted mutagenesis in model species (*Escherichia coli *and *Bacillus subtilis*) and the genetically tractable soil species *Acinetobacter baylyi *[3-5]. Incidentally, this defined the genes necessary to support cellular life (the minimal genome) as those not amenable to mutagenesis. For a few other bacterial species (*Corynebacterium glutamicum*, *Francisella novicida*, *Mycoplasma genitalium*, *Pseudomonas aeruginosa *and *Staphylococcus aureus*) transposon mutagenesis followed by sequencing of the transposon insertion sites has been used to generate large (but incomplete) archived libraries of mutants [6-11]. However, multiple factors often hinder the effectiveness of these toolboxes in contributing to large-scale unraveling of gene function and/or the design of novel therapies, including: slow growth and complex nutritional requirements (*M. genitalium*); the fact that many of these species do not cause disease in humans (*C. glutamicum*, *F. novicida*); the use of strains for which no accurate genome annotation is available; and the frequent lack of publicly accessible online databases for analysis and distribution of the mutants.

*Neisseria meningitidis *(the meningococcus) possesses several features that make it a good candidate among human pathogens for the creation of such a biological resource. The meningococcus, which colonizes the nasopharyngeal mucosa of more than 10% of mankind (usually asymptomatically), grows on simple media with a rapid doubling time and has a relatively compact genome of approximately 2.2 Mbp [12-15]. Furthermore, it is naturally competent throughout its growth cycle and is therefore a workhorse for genetics. Yet, it is a feared human pathogen because, upon entry in the bloodstream, it causes meningitis and/or septicemia, which can be fatal within hours [16]. Each year there are approximately 1.2 million cases of meningococcal infections worldwide, mostly in infants, children and adolescents, leading to an estimated 135,000 deaths [17].

Here we have exploited these meningococcal features to design NeMeSys, a toolbox for *N. meningitidis *systematic functional analysis. We opted for strain 8013 (serogroup C), which was isolated at the Institut Pasteur in 1989 from the blood of a 57-year-old male. This strain belongs to the ST-18 clonal complex, often associated with disease in countries from Central and Eastern Europe. It was chosen primarily because it is well-characterized (extensively used to study adhesion to human cells and type IV pilus (Tfp) biology) and has been previously used to produce an archived library of approximately 4,500 transposon mutants [18]. We created NeMeSys by sequencing the genome of strain 8013, the annotation of which has been performed manually using MicroScope, a powerful platform for microbial genome annotation [19], and sequencing/mapping the transposon insertion sites in 83% of the above mutants, which showed that 924 genes were hit. Taking advantage of *N. meningitidis *natural competence for transformation, we designed a targeted *in vitro *transposon mutagenesis approach useful for completing the library in the future and validated it by constructing 26 mutants. The current library contains mutants in 947 genes of strain 8013. All these datasets were stored in a publicly accessible thematic database (NeisseriaScope) within MicroScope [19]. Furthermore, to maximize the potential of NeMeSys for functional analysis and foster its use in the *Neisseria *community where multiple strains are used, we have manually (re)annotated the following publicly available genome sequences: four *N. meningitidis *clinical isolates from the different clonal complexes MC58 (ST-32, serogroup B), Z2491 (ST-4, serogroup A), FAM18 (ST-11, serogroup C) and 053442 (ST-4821, serogroup C) [12-15]; one unencapsulated *N. meningitidis *carrier isolate (strain α14) [20]; one isolate of the commensal *N. lactamica *(ST-640), which shares the same ecological niche as *N. meningitidis*; and two clinical isolates of the closely related human pathogen *N. gonorrhoeae *(strains FA 1090 and NCCP11945), which colonizes a totally different niche (the urogenital tract) [21]. As above, these genomes have been stored in NeisseriaScope and are publicly accessible. Finally, we present evidence obtained through functional and comparative genomics illustrating how NeMeSys can be used to narrow the gap between sequence and function in the meningococcus.

## Results and discussion

### First component of NeMeSys: the genome sequence of strain 8013

Providing a precise answer to the question of how many genes are present in strain 8013's genome was a key primary task as this is crucial information for the generation of a large collection of defined mutants. We therefore determined the complete genome sequence of this clinical isolate belonging to a clonal complex that is unrelated to the previously sequenced *N. meningitidis *strains [22]. Base-pair 1 of the chromosome was assigned within the putative origin of replication [23]. Unsurprisingly, the new genome displays all the features typical of *N. meningitidis *(Table 1). It contains numerous repetitive elements - which have been extensively studied in other sequenced strains [13,14] - the most abundant of which (1,915 copies) is the DNA uptake sequence essential for natural competence. Although these repeats contribute to genome plasticity, 8013's genome has maintained a high level of colinearity with other *N. meningitidis *genomes. Synteny between 8013's and other meningococcal genomes is either conserved (with α14) or mainly disrupted by single, distinct, symmetric chromosomal inversions (Additional data file 1).

**Table 1:** General features of *N. meningitidis *based on six (re)annotated genome sequences

	*N. meningitidis *strain					
	
Genome feature	8013	Z2491	MC58	FAM18	053442	α14
Size (bp)	2,277,550	2,184,406	2,272,360	2,194,961	2,153,416	2,145,295
G+C content (%)	51.4	51.8	51.5	51.6	51.7	51.9
Coding density (%)	76	76.9	76.5	77.2	76.5	78.3
Genes	1,912	1,878	1,914	1,872	1,817	1,809
Pseudogenes	55	63	69	55	57	59
Truncated genes	69	48	48	56	68	51
Silent cassettes	25	15	24	17	13	10
Strain-specific genes	38	41	37	10	18	44
tRNA	59	58	59	59	59	58
rRNA operons	4	4	4	4	4	4

To achieve an annotation as accurate as possible, we annotated 8013's genome manually by taking advantage of all the functionalities of the MicroScope platform [19]. This previously described annotation pipeline has three main components: numerous embedded software tools and bioinformatics methods for annotation; a web graphical interface (MaGe) for data visualization and exploration; and the large Prokaryotic Genome DataBase (PkDGB) for data storage, which contains more than 400 microbial genomes. We devoted particular care to identifying and duly labeling gene remnants and silent cassettes because these do not encode functional proteins and are, therefore, not targets for mutagenesis. We identified 69 truncated genes (either in 5' or 3'), which we labeled with the prefix 'truncated'. For example, the truncated *rpoN *encodes an inactive RNA polymerase sigma-54 factor with no DNA-binding domain [24]. In addition, there are also three types of putative transcriptionally silent cassettes (25 in total), which we named *tpsS*, *mafS *and *pilS*. These cassettes have an important role in nature, generating antigenic variation upon recombination within the *tpsA *and *mafB *multi-gene families, which encode surface-exposed proteins (but this is yet to be demonstrated) or *pilE*, which encodes the main subunit of Tfp [25,26]. Altogether, 8013's genome contains the information necessary to encode 1,967 proteins. Fifty-five of these proteins are encoded by out of phase genes that we labeled with the suffix 'pseudogene', most of which (94.5%) are inactivated by a single frameshift and are thus present as two consecutive CDSs. Since these pseudogenes result from the slipping of the DNA polymerase through iterative motifs [27], they are usually switched on again during successive rounds of replication (a process known as phase variation) and are, therefore, *bona fide *targets for mutagenesis. As is usual in MicroScope [19], 8013's genome annotation has been stored within PkDGB in a thematic database named NeisseriaScope. To facilitate access to this thematic database, we have designed a simple webpage [28] with direct links to some of the most salient features in MicroScope. Once in MicroScope, the user then has access to a much larger array of exploratory tools [19].

**Figure 1:**
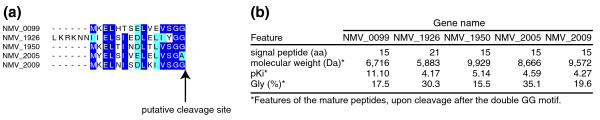
***N. meningitidis *strain 8013 has putative type I secretion units for the export of polypeptides that may play a role in colonization or virulence by acting, respectively, as bacteriocins or pheromones**. **(a) **Alignment of the double-glycine motifs in the putative bacteriocin/pheromones found in strain 8013. Amino acids are shaded in purple (identical) or in light blue (conserved) when present in at least 80% of the aligned sequences. **(b) **General features of the putative bacteriocin/pheromones. aa, amino acids.

The added value of this manual annotation is significant, as illustrated, for example, by the following observation that was previously overlooked. Strain 8013 is very likely to use type I secretion (during which proteins are transported across both membranes in a single step) to export polypeptides that could play a role in pathogenesis. Together with a TolC-like protein forming a channel in the outer membrane (NMV_0625), 8013's genome contains two complete copies of a polypeptide secretion unit consisting of an inner-membrane protein from the ATP-binding cassette ABC-type family, which has a distinctive amino-terminal proteolytic domain of the C39 cysteine peptidase family (NMV_0105/0106 and NMV_1949), an adaptor or membrane fusion protein (NMV_0104 and NMV_1948), and several exported polypeptides with a conserved amino-terminal leader sequence finishing with GG or GA (known as the double-glycine motif) that is processed by the inner-membrane peptidase (Figure 1a). Since double-glycine motifs are not readily identified by bioinformatic methods, we screened the genome of 8013 manually and discovered five candidate genes containing such leader sequences (Figure 1a). The putative mature polypeptides are small, rich in glycine and either very basic or acidic (Figure 1b). Although FAM18 and MC58 strains also contain one complete copy of this secretion unit (while only remnants are found in Z2491 and 053442), this biological information could not be easily extracted from the corresponding genome annotations, in which these genes were predicted to encode proteins of unknown function or to be putative protein export/secretion proteins, at best. What could be the role of these polypeptides, if any, in meningococcal pathogenesis? Although it is more likely that they are bacteriocins [29] with a role in nasopharyngeal colonization through inhibition of the growth of other bacteria competing for the same ecological niche, there is another intriguing possibility. As reported in Gram-positive bacteria, these polypeptides could be pheromones used for quorum sensing and cell-to-cell communication [29]. This possibility is appealing because meningococci are not known to produce other quorum-sensing molecules that could allow them to regulate their own expression profiles in response to changes in bacterial density.

### Second component of NeMeSys: a growing collection of defined mutants in strain 8013

**Figure 2:**
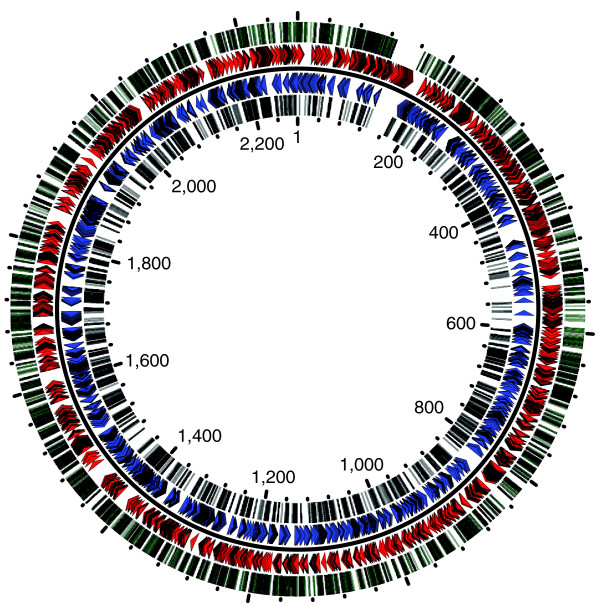
**Distribution on the *N. meningitidis *strain 8013 genome of 3,655 transposon insertions in an archived collection of mutants**. The concentric circles show (reading inwards): insertions in genes (green); genes transcribed in the clockwise direction (red); genes transcribed in the counterclockwise direction (blue); and insertions in intergenic regions (black). Distances are in kbp.

We have previously reported the assembly of an archived library of 4,548 transposition mutants in strain 8013 and the design of a method for high-throughput characterization of transposon insertion sites based on ligation-mediated PCR [18]. Here, we extended this systematic sequencing program to all the mutants in the library and obtained 3,964 sequences of good quality (Table 2). After eliminating 22 sequences for which various anomalies were detected, we kept only one sequence for each mutant (sometimes both sides of the inserted transposon were sequenced); we thus identified the transposon insertion sites in 3,780 mutants (83.1% of the library). Strain 8013's genome sequence made it possible to precisely map 3,625 of these insertions to 3,191 different sites (the remaining 155 being in repeats). This showed that transposition occurred randomly as insertions were scattered around the genome (Figure 2), every 700 bp on average, and no conserved sequence motifs could be detected apart from the known preference for transposition into TA dinucleotides. Strikingly, only 63.4% of the mapped insertions were in genes, which is substantially lower than the 76% coding density of the genome. This bias is likely to be due, at least in part, to the fact that insertions that occurred in essential genes during *in vitro *transposon mutagenesis were counter-selected upon transformation in *N. meningitidis *(see below). Analysis of the insertions within genes shows that a total of 924 genes were hit between 1 and 14 times (Additional data file 2). As expected, larger genes tended to have statistically more hits (Table 3). For example, 86% (24 out of 28) of the genes longer than 3 kbp were hit 5.7 times on average, 62% (58 out of 94) of the genes between 2 and 3 kbp long were hit 3.7 times on average, while only 21% (14 out of 66) of the genes shorter than 200 bp were hit. As above, these data have been stored in NeisseriaScope. Determining whether a gene has been disrupted, how many times, and in which position(s) and requesting the corresponding mutant(s) can therefore easily be done online.

**Table 2:** General features of the collection of defined mutants in strain 8013

Feature	*N*
Random mutagenesis	
Mutants arrayed	4,548
Transposon insertion sites sequenced	3,802
High-quality sequences	3,780
Insertion sites mapped	3,625
Insertions in genes	2,299
Insertions between genes	1,326
Genes hit	924
	
Targeted mutagenesis	
Genes targeted	28
Genes mutated	26
	
Number of genes mutated (total)	947

**Table 3:** Statistical distribution of transposon insertions within genes

Gene size (bp)	Number of genes	% genes hit	Number of hits	Average hits	% genes missed	% missed genes in DEG
≥3,000	28	85.7	137	5.7	14.3	75
2,000-3,000	94	61.7	213	3.7	38.3	77.8
1,000-2,000	557	58.9	954	2.9	41.1	46.7
500-1,000	688	49.5	660	1.9	50.5	41.9
≤500	600	32.7	367	1.9	67.3	31.4

DEG: Database of Essential Genes.

Although the number of essential genes in bacteria vary in different species [30], a likely estimate of 350 genes being essential for growth in *N. meningitidis *suggests that the library contains mutants with insertions in 57.1% of the remaining 1,617 genes that might be amenable to mutagenesis. Although an increase in saturation could be achieved by assembling a much larger library of mutants, this would come at a high cost - that is, a substantial increase in both mutant redundancy and insertions in intergenic regions. We therefore took advantage of 8013's natural competence and strong tendency towards homology-directed recombination to design an alternative targeted mutagenesis strategy, robust enough to be used to complete the library (Table 2). We modified our original mutagenesis method in which genes are amplified, cloned, submitted to *in vitro *transposition and directly transformed in *N. meningitidis *[31] because although it could be used in strain 8013 (we generated mutants in six genes involved in Tfp biology), its efficiency was too variable for high-throughput use. The rationale of the new method was to positively select mutagenized target plasmids in *E. coli *before transforming them into the meningococcus. We therefore subcloned the mini-transposon into a plasmid with a R6K origin of replication that requires the product of the *pir *gene for stable maintenance [32]. This allows positive selection of target plasmids with an inserted transposon in target genes after transformation of the *in vitro *transposition reactions in an *E. coli *strain lacking *pir *(see Materials and methods). As initially shown with *comP *and NMV_0901 (genes with suspected roles in Tfp biology; see below), plasmids suitable for *N. meningitidis *mutagenesis could be readily selected. This method was further validated by constructing 18 mutants in missed genes encoding two-component systems and helix-turn-helix-type transcriptional regulators. Interestingly, although we obtained plasmids suitable for mutagenesis, we could not disrupt *fur*, which encodes a ferric uptake helix-turn-helix-type regulator, and NMV_1818, which encodes the transcriptional regulator of a two-component system (Table 2). At this stage, we have at our disposal a library of mutants in 947 genes of strain 8013 (approximately 60% of the genes that might be amenable to mutagenesis; Table 2), including almost all those involved in Tfp biology and transcriptional regulation, and a robust mutagenesis method for completing it in the future.

### Functional genomics: NeMeSys facilitates identification of gene function and genes essential for viability

**Figure 3:**
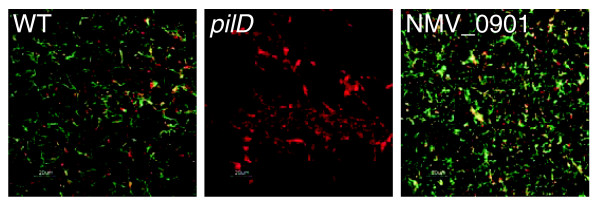
**NMV_0901 is not involved in Tfp biogenesis**. Presence or absence of Tfp in various genetic backgrounds as monitored by immunofluorescence microscopy. Fibers were stained with a pilin-specific monoclonal antibody (green) and the bacteria were stained with ethidium bromide (red).

The main aim of NeMeSys is to facilitate identification of gene function, notably the discovery of genes essential for meningococcal pathogenesis and/or viability. The potential of NeMeSys for discovery of genes essential for pathogenesis has already been confirmed by the results of several screens performed at earlier stages of the construction of this resource. These studies improved our understanding of properties key for meningococcal virulence, such as resistance to complement-mediated lysis [18], adhesion to human cells [33] or Tfp biogenesis [34]. For example, we previously showed that 15 genes are necessary for Tfp biogenesis (*pilC1 *or *pilC2*, *pilD*, *pilE*, *pilF*, *pilG*, *pilH*, *pilI*, *pilJ*, *pilK*, *pilM*, *pilN*, *pilO*, *pilP*, *pilQ *and *pilW*) as the corresponding mutants are non-piliated [34]. To further strengthen this point, we decided to revisit, using the current version of NeMeSys, our findings on Tfp biogenesis that made *N. meningitidis *strain 8013 a model for the study of this widespread colonization factor [35]. Firstly, we noticed that the original screen was extremely efficient because approximately 96% of the mutants in these genes that are present in the library (47 out of 49) were indeed identified. Secondly, mining of 8013's genome uncovered 8 additional genes for which their sequence (*pilT2*) and/or previous reports (*comP*, *pilT*, *pilU*, *pilV*, *pilX*, *pilZ *and NMV_0901) suggest that they could play a role in Tfp biology. Although most of these genes have been studied in other piliated species, their role in piliation is not always clear as conflicting phenotypes have been assigned to some of the corresponding mutants [35]. Therefore, after constructing the corresponding mutants (50% of these genes were not mutated in the original library), we used immunofluorescence microscopy to visualize Tfp. This demonstrated that none of these genes is necessary for Tfp biogenesis in *N. meningitidis*. Importantly, mutants in NMV_0901 are unambiguously piliated (Figure 3) despite its annotation as a putative fimbrial assembly protein in every bacterial genome where it is present, including the previously published *N. meningitidis *genomes. Strikingly, this annotation was inferred only from sequence homology with FimB from *Dichelobacter nodosus*, which was once hypothesized to be involved in Tfp biogenesis [36], a possibility that was later invalidated [37]. Our results confirm that the annotation of this CDS should, therefore, be updated in the databanks and in future genome projects.

Essential genes are defined as those not amenable to mutagenesis. During targeted mutagenesis, the absence of transformants with plasmids generated by the above method is strong evidence that the corresponding genes are essential since transformation of strain 8013 with plasmids is usually very efficient (up to 1,000 transformants per microgram of DNA). For example, although we obtained plasmids suitable for mutagenesis, we could not obtain mutants in *fur *and NMV_1818, which suggests that these genes are essential, at least in strain 8013. Furthermore, genes without transposon insertions that are almost certainly essential could readily be highlighted by a statistic analysis. For example, we found that non-repeated genomic regions devoid of transposons that are significantly larger than the average distance between insertions (700 bp) predominantly contain genes listed in the Database of Essential Genes (DEG) [30]. DEG, which lists bacterial genes essential for viability in different species, has therefore been integrated into MicroScope to facilitate this analysis. This is best illustrated by the largest such region (Figure 2), which starts at 130,211, is 36.6 kbp long, and contains 47 genes but not a single tranposon insertion. At least 44 of these genes are almost certainly essential according to DEG, such as the 32 genes that encode protein components of the ribosome. Similarly, this holds true for most of the large genes that were missed (Table 3). Of the four genes longer than 3 kbp that were missed, three are almost certainly essential (*rpoC*, *rpoB *and *dnaE*) as they are involved in basic RNA and DNA metabolism. Of the 36 genes between 2 and 3 kbp long that were missed, approximately 80% are almost certainly essential, such as those encoding 7 tRNA-synthetases or proteins involved in DNA metabolism (*dnaZ/X*, *ligA*, *gyrA*, *gyrB*, *nrdA*, *parC*, *pnp*, *priA*, *rne*, *topA *and *uvrD*). Interestingly, not all genes listed in DEG are essential in the meningococcus, as we found insertions in *ftsE *and *ftsX *(involved in cell division), which are essential in *E. coli*, or *fba *(fructose-bisphosphate aldolase), which is essential in *P. aeruginosa*. This points to interesting differences between *N. meningitidis *and these species.

### Third component of NeMeSys: eight additional (re)annotated Neisseria genomes

To facilitate and foster the use of NeMeSys in the *Neisseria *community where multiple strains are used, we have included in NeisseriaScope all the publicly available complete *Neisseria *genomes (five *N. meningitidis*, two *N. gonorrhoeae *and one *N. lactamica*). However, we noticed that the annotations (*N. lactamica *is not annotated yet) were heterogeneous, which probably results from the use of different CDS prediction software and/or different annotation criteria. We have therefore (re)annotated each genome in MicroScope. In brief, we first transferred 8013's gene annotation to the clear orthologs in these genomes (CDSs identified by BLASTP as encoding proteins with at least 90% amino acid identity over at least 80% of their length). We then manually edited the annotation of the remaining CDSs in Z2491 using the criteria set for strain 8013 and transferred this annotation to the remaining genomes using the same cutoff. This was then done iteratively in the order MC58, FAM18, 053442, *N. lactamica*, FA 1090, NCCP11945 and α14. An additional approximately 4,000 CDSs have thus been manually curated, bringing the grand total to approximately 6,400 (Table 4). As above, all these datasets are stored in NeisseriaScope and are readily accessible online. During this process, we deleted as many as 1,238 previously predicted CDSs (43% of which are in NCCP11945 only), mostly (85%) because they were not identified as CDSs by MicroScope (Table 4). The possibility that most of these CDSs were actually prediction errors is strengthened by two facts. Firstly, despite the corresponding genomic regions being often conserved in all genomes, as revealed by BLASTN, these CDSs were originally predicted in only one or two genomes. Secondly, they were occasionally replaced in other genomes by overlapping correct CDSs on the opposite strand. Among the many such examples are NMB0936 in MC58 (wrong) replaced by NMA1131 and NMA1132 in Z2491 (correct), and NMCC_1055 in 053442 (wrong) replaced by NMC1074 in FAM18 (correct). In parallel, we added 912 new CDSs (Table 4). For example, clearly missing in the original annotations were genes as important as *tatA/E *in FAM18, which encodes the TatA/E component of the Sec-independent protein translocase, *ccoQ *in MC58, which encodes one of the components of cytochrome c oxidase, and as many as eight genes encoding ribosomal proteins in NCCP11945. By improving homogeneity of the *Neisseria *genome annotations, this massive effort is expected to have an impact on future studies aimed at narrowing the gap between sequence and function in these species.

**Table 4:** Summary of the (re)annotation effort of eight *Neisseria *genomes

	Strain							
	
Genome feature	Z2491	MC58	FAM18	053442	N. lactamica*	FA 1090	NCCP11945	α14
Manually edited CDSs	466	421	315	574	549	643	691	314
CDSs deleted from previous annotation	103	164	39	138		173	538	83
New CDSs	38	93	91	100		362	150	78

*The *N. lactamica *genome was not previously annotated.

### Comparative genomics: NeMeSys facilitates whole-genome comparisons

**Figure 4:**
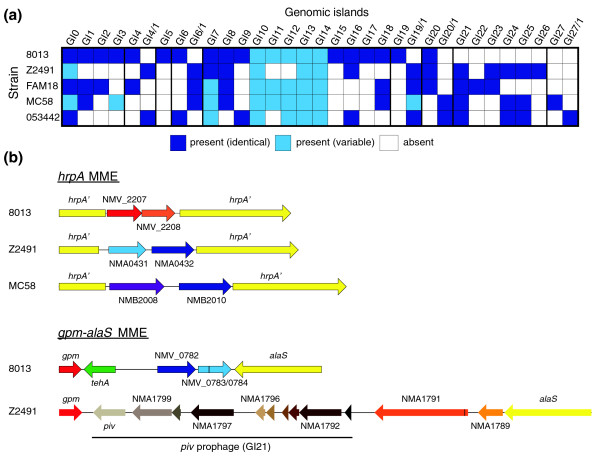
**Most non-core meningococcal genes are clustered in approximately 20 genomic islands (GIs) in a limited number of genomic regions**. **(a) **Presence and distribution of GIs possibly acquired by horizontal transfer (see Additional data file 4 for a detailed list of genes in the GIs). **(b) **Novel genomic context of some minimal mobile elements (MME), regions of high plasticity occupied by different GIs in different strains. Genes of the same color encode orthologous proteins. All the genes are drawn to scale.

Whole-genome comparisons, *in silico *or using microarrays, have been widely used to gain novel insight into the biology of *Neisseria *species [22,38-41]. The availability of nine homogeneously (re)annotated *Neisseria *genomes is expected to facilitate comparative genomics, notably by preventing some erroneously predicted CDSs from appearing as strain-specific and by increasing the number of genes common to all strains. A basic analysis of *N. meningitidis *strains revealed extremely conserved features (Table 1) and provided the identikit of a typical meningococcus. The theoretical average meningococcal genome is 2.2 Mbp long and contains the information necessary to encode 1,927 proteins (truncated genes and silent cassettes are excluded from this count). Each strain contains, on average, 31 genes showing no homology to genes present in the other genomes (Table 1), confirming recent predictions [38] that the pan-genome of *N. meningitidis *(the entire gene repertoire accessible to this species [42]) is open and large. A comparison of *N. meningitidis *clinical isolates (all strains except α14) shows that as many as 1,736 genes (approximately 90%) are shared (Additional data file 3) since they encode proteins displaying at least 30% amino acid identity over at least 80% of their length and are, in addition, in synteny and/or are bidirectional best BLASTP hits (BBHs). Importantly, this number is only slightly decreased when changing the cutoff to a very stringent 80% amino acid identity (data not shown). This shows that despite its fundamentally non-clonal population structure, *N. meningitidis *is more homogeneous than predicted using previous annotations [22]. Nevertheless, the potential for diversity is important and results from the presence of approximately 200 non-core genes (approximately 10% of the gene content). In each genome, many of these non-core genes cluster together in approximately 20 genomic islands (GIs), most of which are likely to have been acquired by horizontal transfer (Figure 4a). These GIs, many of which were previously identified in other genomes as prophages, composite transposons or so-called minimal mobile elements [22,43,44], contain *maf *and *tps *genes, genes involved in the biosynthesis of the capsule or the secretion of bacteriocin/pheromones, and genes encoding FrpA/C proteins or type I, II and III restriction systems (Additional data file 4). Interestingly, identification of novel combinations of non-core genes flanked by core genes - for example, those defining GI19 and GI20 (Figure 4b) - provide further evidence for the minimal mobile element model in which these units promote diversity through horizontal gene transfer and chromosomal insertion by homologous recombination [44]. In conclusion, the fact that approximately 90% of meningococcal genes are conserved in clinical isolates is a clear advantage for NeMeSys as it indicates that a complete library of mutants in strain 8013 could be used to define the functions of most genes in any *N. meningitidis *strain.

**Figure 5:**
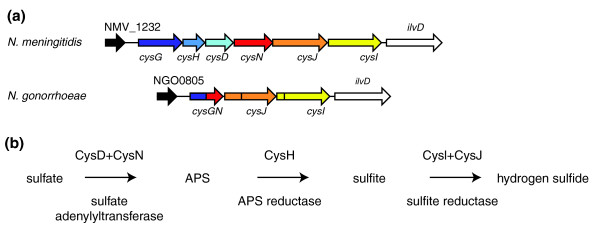
***Neisseria *species colonizing the human nasopharynx (*N. meningitidis *and *N. lactamica*), but not *N. gonorrhoeae*, which colonizes the genital tract, have a complete metabolic pathway potentially involved in sulfate reduction**. **(a) **Genomic context of the genes likely to be involved in sulfate reduction in *N. meningitidis *(identical in *N. lactamica*) and in *N. gonorrhoeae*. Genes of the same color encode orthologous proteins. *cysI *and *cysJ *in the gonococcus are pseudogenes and the frameshifts are represented by horizontal lines within the CDS. All the genes are drawn to scale. **(b) **Predicted biochemical pathway for sulfate reduction in *N. meningitidis*. APS: adenosine 5'-phosphosulfate.

Examination of the core genome confirms well-known facts [12-15], such as that *N. meningitidis *has a robust metabolism (complete sets of enzymes for glycolysis, the tricarboxylic acid cycle, gluconeogenesis and both pentose-phosphate and Entner-Doudoroff pathways) and may be capable of *de novo *synthesis of all 20 amino acids. Inspection of the non-core genome outlines differences between clinical isolates that might modulate their virulence, such as a truncated *pilE *gene in 053442, which suggests that this strain is non-piliated and has impaired adhesive abilities, or the presence of the hemoglobin-haptoglobin utilization system HpuA/B [45], which might improve the ability of FAM18 and Z2491 to scavenge iron in the host. However, to illustrate NeMeSys's utility for comparative genomics, rather than trying to identify genes important for meningococcal pathogenesis, which is elusive since several studies have shown that putative virulence genes are found in both clinical isolates and non-pathogenic strains or species such as *N. meningitidis *α14 and *N. lactamica *[38,40,41], we looked for 'fitness' genes that might be important for nasopharyngeal colonization. To do this we identified the genes shared by all *N. meningitidis *and *N. lactamica *strains (encoding proteins displaying at least 50% amino acid identity over at least 80% of their length and are, in addition, in synteny and/or BBHs) and absent in the two gonococci (which colonize the urogenital tract). This led to an intriguing novel finding. Out of the only nine genes present in the seven nasopharynx colonizers but missing in the two genital tract colonizers (Table 5), three (*cysD*, *cysH *and *cysN*) encode proteins that are part of a well-characterized metabolic pathway. In *N. gonorrhoeae*, an in-frame 3.4 kbp deletion has occurred between *cysG *and *cysN*, leading to a gene encoding a composite protein of which the amino-terminal half corresponds to the amino-terminal approximately 34% of CysG and the carboxy-terminal half corresponds to the carboxy-terminal approximately 45% of CysG (Figure 5a). In *N. meningitidis *and *N. lactamica*, the five proteins encoded by *cysD*, *cysH*, *cysI*, *cysJ *and *cysN *are expected to give these species the ability to reduce sulfate into hydrogen sulfide (Figure 5b). First, CysD and CysN might transform sulfate into adenosine 5'-phosphosulfate (APS). Usually, APS is phosphorylated into phosphoadenosine-5'-phosphosulfate (PAPS), which is then reduced into sulfite by a PAPS reductase, but there is no gene encoding the necessary enzyme (APS kinase). This might have led to the conclusion that the pathway is incomplete. However, unlike what has been predicted in previous annotations, the product of *cysH *is likely to be a PAPS reductase rather than an APS reductase since it is most closely related to genes encoding APS reductases in alphaproteobacteria such as *Sinorhizobium meliloti *and *Agrobacterium tumefaciens *and plants such as *Arabidopsis thaliana *[46]. Therefore, in *N. meningitidis *and *N. lactamica *sulfate reduction differs slightly from the classical pathway since APS might be directly reduced into sulfite by CysH (Figure 5b). The possibility that sulfur metabolism might be critical for meningococcal survival in the host, which remains to be experimentally tested, is not unprecedented in bacterial pathogens, as shown in *Mycobacterium tuberculosis *[47].

**Table 5:** Genes shared by six *N. meningitidis *strains and *N. lactamica *that are absent in two *N. gonorrhoeae *strains, some of which may play a role in nasopharyngeal colonization

Label	Gene	Product
NMV_1014		Conserved hypothetical protein
NMV_1017		Hypothetical protein
NMV_1172/1173		Putative glycosyl transferase (pseudogene)
NMV_1233	*cysG*	Siroheme synthase
NMV_1234	*cysH*	Adenosine phosphosulfate reductase (APS reductase)
NMV_1235	*cysD*	Sulfate adenylyltransferase small subunit
NMV_1236	*cysN*	Sulfate adenylyltransferase large subunit
NMV_2185		Conserved hypothetical integral membrane protein
NMV_2186		Hypothetical membrane-associated protein

These genes, which are in synteny and/or are BBHs, encode proteins displaying at least 50% amino acid identity over at least 80% of their length.

## Conclusions

We have designed a biological resource for large-scale functional studies in *N. meningitidis *that, as illustrated here, has the potential to rapidly improve our global understanding of this human pathogen by promoting and facilitating functional and comparative genomics studies. NeMeSys is viewed as an evolving resource that will be improved, for example, through completion of the collection of mutants (either through gene-by-gene or systematic targeted mutagenesis of the missed genes), further improvement of the accuracy of the annotation by taking into account any new experimental evidence, improvement of the website design and content, and addition of new *Neisseria *genomes as they become available. There is no doubt that NeMeSys would requite these efforts (thereby justifying its name, which was inspired by an ancient Greek goddess seen as the spirit of divine retribution) by further improving our capacity to understand gene function in *N. meningitidis*. Ideally, such studies could contribute to the ongoing efforts aimed at comprehensively understanding a prokaryotic cell and help in the design of new therapies.

## Materials and methods

### Bacterial strains and growth conditions

The sequenced strain (also known as clone 12 or 2C43) is a naturally occurring pilin antigenic variant of the original clinical isolate *N. meningitidis *8013, which expresses a pilin mediating better adherence to human cells [48]. Meningococci were grown at 37°C in a moist atmosphere containing 5% CO_2 _on GCB agar plates containing Kellog's supplements and, when required, 100 μg/ml kanamycin. *E. coli *TOP10 (Invitrogen, Paisley, Renfrewshire, UK), DH5α or DH5α λ*pir *were grown at 37°C in liquid or solid Luria-Bertani medium (Difco, Oxford, Oxfordshire, UK), which contained 100 μg/ml ampicillin, 100 μg/ml spectinomycin and/or 50 μg/ml kanamycin, when appropriate.

### Genome sequencing

The complete genome sequence of strain 8013 [EMBL:FM999788] was determined by a whole genome shotgun using a library of small inserts in pcDNA 2.1 (Invitrogen). We obtained and assembled 32,338 sequences using dye-terminator chemistry, which gave an approximately nine-fold coverage of the genome. End sequencing of large inserts in a pBeloBAC11 library aided in assembly verification and scaffolding of contigs.

### Genome (re)annotations

Strain 8013's genome was annotated using the previously described MicroScope annotation pipeline [19], which has embedded software for syntactic analysis and more than 20 well-known bioinformatics methods (InterProScan, COGnitor, PRIAM, tmHMM, SignalP, and so on). In brief, potential CDSs were first predicted by the AMIGene software [49] using three specific gene models identified by codon usage analysis, tRNA were identified using tRNAscan-SE [50], rRNA using RNAmmer [51] and other RNA by scanning the Rfam database [52]. CDSs were assigned a unique NMV_ identifier and were submitted to automatic functional annotation in MicroScope [19]. Functional annotation, syntactic homogeneity and start codon position of each CDS present in the genome were then refined manually during three rounds of inspection of the results obtained using the above bioinformatics methods. This led to four major classes: CDSs encoding proteins of known function (high homology to proteins of defined function), for which the SwissProt annotation was most often used; CDSs encoding proteins of putative function (conserved protein motif/structural features or limited homology to proteins of defined function), which were labeled with the prefix 'putative'; and CDSs encoding proteins of unknown function defined either as 'conserved hypothetical protein' (significant homology to proteins of unkown function outside of *Neisseria *species) or 'hypothetical protein' (no significant homology outside of *Neisseria *species). However, adjectives were added when localization of the corresponding proteins could be predicted through tmHMM [53] or SignalP [54] (for example, 'hypothetical periplasmic protein' or 'conserved hypothetical integral membrane protein') or protein motifs not allowing functional predictions were identified through InterProScan [55] (for example, 'conserved hypothetical TPR-containing protein'). Importantly, during the manual curation of CDSs encoding proteins of unknown function, the dubious ones (typically those with less than 50% coding probability, shorter than 150 bp, overlapping with highly probable CDSs or RNA on the opposite strand, and so on) were deleted. During this process, self-explanatory comments mostly based on InterProScan entries and links to relevant literature in PubMed (139 in total) were entered manually in the database.

To define truncated genes, for which only partial homologies could be detected, or out of phase genes, for which homology was complete but involved at least two consecutive CDSs, we used BLASTP and coding probability results. The corresponding open reading frames were trimmed to their biologically significant portions (both on 5' and 3') and labeled with the prefix 'truncated' or the suffix 'pseudogene', respectively. During this process, putative frameshifts or sequencing errors in 42 CDSs were amplified and resequenced.

All *Neisseria *genomes available in GenBank (MC58, Z2491, FAM18, 053442, α14, FA 1090 and NCCP11945) or at the Sanger Institute (*N. lactamica*) were (re)annotated in MicroScope using the same approach as above. AMIGene was used to predict the CDSs, labeling the new ones with a distinct identifier (for example, NEIMA instead of NMA in Z2491), which were submitted to automatic functional annotation in MicroScope. The functional annotation in *N. meningitidis *strain 8013 was then automatically transferred to all clear orthologs, stringently defined as genes endoding proteins showing at least 90% BLASTP identity over at least 80% of their length. All the remaining CDSs were then annotated manually using the same procedure as for strain 8013, starting with Z2491 and transferring this new annotation to the remaining genomes using the same cutoff. This was then done iteratively in the order MC58, FAM18, 053442, *N. lactamica*, FA 1090, NCCP11945 and α14. Importantly, previously predicted CDSs that were not recognized as such by AMIGene were deleted during the process.

### Genomic analyses

All the genomic analyses were performed within MicroScope using embedded software. Whole-genome comparisons of gene content (using the mentioned cutoffs) were done using the PhyloProfile Synteny functionality [19], which combines BLASTP, BBH and/or synteny results. Graphical representation of whole-genome syntenies were generated using LinePlot functionality [19]. Graphical circular representation of the strain 8013 genome with transposon insertions was generated using the CGView software [56]. Characterization of the sulfate reduction pathway in *Neisseria *strains colonizing the nasopharynx was done using metabolic pathway predictions built with the Pathway Tools software [57]. GIs of putative horizontally transferred genes were identified in each *N. meningitidis *clinical isolate using the Genomic Island functionality tool [19]. This tool combines detection of synteny break points in the query genome in comparison with closely related genomes, searches for mobility genes, tRNA and direct repeats (if any) at the borders of the synteny break points and finally searches for compositional bias in the query genome.

### Genome-wide collection of defined mutants

The construction of an archived library of undefined transposon mutants in strain 8013 and the design/validation of a method for large-scale characterization of transposon insertion sites based on ligation-mediated PCR have been described [18]. Each mutant is assigned a unique x/y identifier, where x indicates the half microtitre plate and y the position of the mutant. Genomic DNA for each mutant, prepared using the Wizard Genomic DNA Purification kit (Promega, Southampton, Hampshire, UK), was used to try to amplify sequences flanking the inserted transposons mainly by ligation-mediated PCR (other techniques have been tested as well). Amplified fragments were sequenced with outward-reading primers ISL or ISR internal to the transposon [18]. Sequences were trimmed to eliminate regions of poor quality or corresponding to the transposon and subsequently mapped on 8013's genome using BLASTN.

Additional mutants were engineered by *in vitro *transposon mutagenesis on PCR products cloned into pCRII-TOPO or pCR8/GW/TOPO vectors (both from Invitrogen). Initially, mutants in six genes involved in Tfp biology (*pilM*, *pilN*, *pilO*, *pilT*, *pilU *and *pilZ*), four of which have been described previously [58], were constructed by directly transforming transposition reactions into strain 8013. We used as a donor the pSM1 vector in which the transposon is cloned within a plasmid with a ColE1 origin of replication [31]. However, the efficiency was low, with only zero to two mutants per transposition reaction. Subsequently, we modified this method for high-throughput use by subcloning the mini-transposon into plasmid pGP704, which has a R6K origin of replication. The mini-transposon, extracted from pSM1 on a *Xba*I-*Eco*RI fragment, was cloned into *Xba*I-*Eco*RI-cut plasmid pGP704 [32]. The resulting plasmid pYU29 can replicate only in the presence of Pir, which is found in *E. coli *strains such as DH5α λ*pir*. Therefore, upon transformation of an aliquot of the *in vitro *transposition reaction in DH5α and selection on plates containing kanamycin (cassette in the mini-transposon) and spectinomycin (cassette in the target vector), target plasmids with an inserted mini-transposon can be positively selected. As seen initially with the *comP *and NMV_0901 genes, hundreds of Sp^r^, Km^r ^transformants could easily be obtained while no transformants were obtained when no transposase was added in the transposition reaction (data not shown). Restriction analysis of recombinant plasmids confirmed that they contained an inserted transposon (data not shown). Transformants containing plasmids suitable for *N. meningitidis *mutagenesis - that is, with an insertion approximately in the middle of the target gene - were readily identified by colony-PCR by using a mix of ISL and ISR, and the forward primer used to amplify the target gene. Plasmids were then extracted, used to sequence the site of transposon insertion with ISL or ISR, and transformed in *N. meningitidis*. This method was validated by constructing mutants in 20 genes (NMV_0125, NMV_0126, NMV_0323, NMV_0419, NMV_0433, *mtrR*, NMV_0658, NMV_0757, NMV_0773, NMV_0774, NMV_0901, *hexR*, *iscR*, NMV_1093, NMV_1134, NMV_1850, NMV_2068, NMV_2160, *comP *and NMV_2258).

### Tfp detection

Tfps were detected by immunofluorescence microscopy using the 20D9 monoclonal antibody, which is specific for the pilin in strain 8013 as described elsewhere [34]. This was done using a Nikon Eclipse E600 microscope and digital images were recorded with a Nikon DXM1200 digital camera mounted onto the microscope.

### Data sharing

As usual in MicroScope [19], all the datasets generated during this study have been stored within PkDGB in a thematic sub-database named NeisseriaScope, which is publicly accessible through MaGe. The MaGe web interface can be used to visualize genomes (simultaneously with synteny maps in other microbial genomes, one of its main features), perform queries (by BLAST or keyword searches) and download all datasets in a variety of formats (including EMBL and GenBank). However, to facilitate access to the genome (re)annotations and distribution of mutants to the scientific community, we have designed a straightforward webpage [28] providing direct links to some of the most salient features in MicroScope. If needed, once in NeisseriaScope, the user has unlimited access to the whole array of exploratory tools within the MicroScope platform. Eventually, upon completion, the library of mutants will be made entirely and freely available. In the meantime, up to ten mutants can be requested simultaneously.

## Abbreviations

APS: adenosine 5'-phosphosulfate: BBH: bi-directional best BLASTP hit; CDS: coding sequence; DEG: Database of Essential Genes; GI: genomic island; PAPS: phosphoadenosine-5'-phosphosulfate; PkDGB: Prokaryotic Genome DataBase; Tfp: type IV pilus.

## Authors' contributions

CR, CB, PG and VP sequenced and assembled strain 8013's genome. DV, AL and CM contributed and managed bioinformatics resources. DV and VP performed manual annotation and bioinformatics analyses. SF and CMS sequenced transposon insertion sites in the library of mutants. HE and VP constructed mutants by targeted mutagenesis. DB and VP performed the functional characterization of Tfp biogenesis. VP conceived the study and was responsible for its coordination. CR, DV, CB, CM, PG and VP wrote the paper.

## Additional data files

The following additional data are available with the online version of this paper: a figure showing global pairwise genome syntenies between strain 8013 and each sequenced *N. meningitidis *strain (Additional data file 1); a table listing genes in strain 8013 that have been disrupted in the collection of mutants (Additional data file 2); a table listing genes shared by all *N. meningitidis *clinical isolates (Additional data file 3); a table listing the genomic islands in each *N. meningitidis *clinical isolate likely to have been acquired by horizontal transfer (Additional data file 4).

## Supplementary Material

Additional data file 1 Strand conservation is indicated in purple, while strand inversions (because of chromosomal inversions) are in blue. Except in strain α14 where there is conserved colinearity, synteny is mainly disrupted by a single chromosomal inversion between the two genes that are indicated. For the 8013/Z2491 comparison, the readout is more difficult because the start of the Z2491 genome was not assigned at the origin of replication. Plots were generated using the LinePlot program within MicroScope after setting the minimum synton size to 40 genes. Click here for file

Additional data file 2 Genes disrupted through targeted mutagenesis are shaded in light blue. Click here for file

Additional data file 3 These genes, which are in synteny and/or are BBHs, encode proteins displaying at least 30% amino acid identity over at least 80% of their length. Click here for file

Additional data file 4 For each GI, the first and the last gene are indicated, together with a comment about their putative roles. Click here for file
